# Expression of cytokine‐induced neutrophil chemoattractant suppresses tumor necrosis factor alpha expression and thereby prevents the follicles from undergoing atresia and apoptosis

**DOI:** 10.1002/rmb2.12022

**Published:** 2017-03-12

**Authors:** Yu Tanaka, Akira Kuwahara, Kenjiro Ushigoe, Yuya Yano, Yuka Taniguchi, Yuri Yamamoto, Toshiya Matsuzaki, Toshiyuki Yasui, Minoru Irahara

**Affiliations:** ^1^ Department of Obstetrics and Gynecology Japanese Red Cross Kochi Hospital Kochi Japan; ^2^ Department of Obstetrics and Gynecology Institute of Health Biosciences The University of Tokushima Graduate School Tokushima Japan

**Keywords:** apoptosis, follicle, ovary, ovulation, tumor necrosis factor alpha

## Abstract

**Aim:**

Cytokine‐induced neutrophil chemoattractant (CINC/gro) is a CXC family chemokine, similar to interleukin‐8 in rats, and is one of the factors that regulates ovulation. However, the mechanism that regulates atresia of the ovaries postovulation is not clearly defined.

**Methods:**

Whether antibody‐blocking of CINC/gro can alter the number of ovulated oocytes and modulate neutrophil infiltration was investigated. The effect of the antibody on the level of inflammatory cytokine production and follicular atresia was examined. Apoptosis was measured by the terminal deoxynucleotidyl transferase‐mediated dUTP nick end labeling (TUNEL) method and via analysis of the messenger RNA expression of Bcl‐2 and Bcl2‐associated X (Bax).

**Results:**

The anti‐CINC/gro antibody treatment decreased the number of ovulated oocytes. The messenger RNA levels of cyclooxygenase‐2 and interleukin‐1 beta were decreased by the antibody treatment, whereas that of tumor necrosis factor (TNF) alpha was increased. The TUNEL analysis revealed a larger number of apoptotic cells in the antibody group, compared with those in the control group, as well as a significant increase in the Bax/Bcl‐2 ratio 24 hours after human chorionic gonadotropin administration.

**Conclusion:**

These findings suggest that ovulation is accelerated by neutrophil infiltration into the theca layer. The CINC/gro appears to synergize with interleukin‐1 beta for ovulation. By contrast, the data suggest that CINC/gro expression suppresses TNF alpha expression and that CINC/gro expression therefore prevents the follicles from undergoing atresia and apoptosis.

## 1 Introduction

The cytokine‐induced neutrophil chemoattractant (CINC/gro) belongs to the CXC family of chemokines, along with interleukin (IL)‐8, and functions as a neutrophil chemoattractant.^1^ The IL‐8 family plays an important role at inflammatory sites, which are sites of leukocyte infiltration.^2^, ^3^

The ovulatory process resembles an inflammatory reaction because, during this process, neutrophils infiltrate into the theca layer of the ovaries.^4^, ^5^, ^6^ It has been shown that neutrophils accumulate in the theca layer of the ovaries in humans and rats just prior to ovulation and that the expression of IL‐8 and CINC/gro, respectively, are responsible for this accumulation.^4^, ^7^, ^8^ Earlier reports have shown that IL‐8 and CINC/gro production are enhanced by human chorionic gonadotropin (hCG).^5^, ^9^, ^10^ It also has been reported that CINC/gro production is increased by the addition of IL‐1 beta and tumor necrosis factor (TNF) alpha to cultures of whole ovarian dispersates.^5^

It is well known that cyclooxygenase (COX)‐2 is essential for ovulation. There are two isoforms of COX: COX‐1 and COX‐2.^11^ COX‐1 is a constitutively active isoenzyme in many cells and keeps cellular homeostasis. In contrast, COX‐2 is an inducible isoform that is responsive to many growth factors and to cytokine stimulation, especially to the factors that are produced during ovulation.^11^, ^12^ The ovulation rate is reduced by COX‐2 suppression^13^, ^14^ and this suppression can be inhibited by supplying prostaglandin E_2_ or IL‐1 beta.^13^, ^15^

Although ovulation has been well studied, the system of luteinization and atresia of the ovaries postovulation is not clearly understood. It has been reported that apoptosis is one of the important elements for luteinization and atresia.^16^, ^17^, ^18^ Apoptosis is controlled by the expression of a number of regulatory genes, such as the genes that encode the Bcl‐2 family.^19^, ^20^ Bcl‐2 is known to protect cells from apoptosis, whereas Bcl2‐associated X (Bax), which also belongs to the Bcl‐2 family, induces apoptosis when present as a homodimer.^20^ However, Bcl‐2 can inhibit Bax‐induced apoptosis by forming heterodimers with Bax.^21^ Thus, the ratio of Bcl‐2 and Bax expression is considered to be important for the regulation of cellular apoptosis.^22^, ^23^, ^24^

In the present study, the function of CIN/gro in ovulation, particularly postovulation, was investigated by assaying the effect of the antibody‐blocking of CINC/gro on the number of ovulated oocytes. It also was determined how the blocking of CINC/gro might modulate luteinization and atresia of the follicles, as well as the production of inflammatory cytokines (IL‐1 beta and TNF alpha) and of COX‐2, and how it might affect apoptosis of the follicles. Apoptosis was assessed by using the terminal deoxynucleotidyl transferase (TdT)‐mediated dUTP nick end labeling (TUNEL) method and by analysis of the messenger RNA (mRNA) expression of Bcl‐2 and Bax.

## 2 Materials and Methods

### 2.1 Reagents

Modified human tubal fluid (mHTF) medium was obtained from Irvine Scientific (Santa Ana, CA, USA) and pregnant mare serum gonadotropin (PMSG) and hCG were obtained from Sigma Chemical Company (St. Louis, MO, USA). The rabbit anti‐rat CINC/gro‐1 antibody was purchased from Immuno‐Biological Laboratories Company (Fujioka, Japan), while the rabbit anti‐rat COX‐2 (N‐20) and IL‐1 beta (H‐153) antibodies and goat anti‐rat TNF alpha antibody (N‐19) were purchased from Santa Cruz Biotechnology (Santa Cruz, CA, USA). All of these are monoclonal antibodies.

### 2.2 Animals and the superovulation procedure

All the experiments were conducted in accordance with the ethical standards that had been established by the institutional animal care and use committee of the University of Tokushima, Tokushima, Japan. Immature (21 day old) female Sprague–Dawley rats were obtained from Charles River Japan (Yokohama, Japan). The rats were induced to superovulate by an intraperitoneal injection of 10 IU of PMSG, followed by 10 IU of hCG 48 hours later. At the same time as the hCG injection, 10 μg of anti‐rat CINC/gro antibody was administered (antibody group). Simultaneously, normal serum was administered to the control rats (control group). In this model, ovulation occurs between 12 and 15 hours after the hCG injection.^25^ The above procedure represents a well‐established and commonly reported model that is used for the study of rat ovulation mechanisms. This method enabled the obtaining of samples in a more timely fashion than by using adult female rats with spontaneous estrous cycles. The rats were killed by cervical dislocation 6, 12, and 24 hours after the hCG injection. At each time point, the unilateral side ovaries were removed immediately and bathed in RNA stabilization solution (RNAlater; Ambion, Austin, TX, USA) and were stored in RNAlater at −20°C until their use. The ovaries on the other side were fixed in 4% paraformaldehyde at 4°C overnight and then were embedded in paraffin.

### 2.3 Number of oocytes

The total number of ovulated oocytes was counted. The oviducts of the rats were removed at times later than 24 hours after the hCG injection. The ovulated cumulus–oocyte complexes were removed from the oviducts and the cumulus–corona complex then was removed by soaking it in mHTF that contained 32 IU/mL of hyaluronidase in order to facilitate the counting of the oocytes.

### 2.4 Neutrophil infiltration

In order to examine neutrophil infiltration, tissue samples from the rats were histologically stained 6, 12, and 24 hours after the hCG injection. The ovaries that had been embedded in paraffin were sectioned (2 μm thick). The prepared sections were stained with periodic acid–Schiff (PAS) to identify neutrophilic elastase. Briefly, the ovarian sections were deparaffinized in xylene and dehydrated in a graded series of ethanol. The slides then were oxidized in 0.5% periodic acid for 10 minutes and washed in running water for 5 minutes. After staining in Schiff's reagent for 20 minutes, the slides were rinsed three times in sodium metabisulfite, washed in running water for 5 minutes, and counterstained with hematoxylin. Subsequently, the slides were mounted following dehydration in ethanol and xylene.

### 2.5 Total RNA extraction and reverse transcription

The frozen ovaries were cut and weighed. The total RNA was extracted from the ovaries by using the RNeasy Protect Mini Kit (Qiagen, Hilden, Germany), according to the manufacturer's instructions. The RNA was dissolved in water and was spectroscopically quantified at 260 nm. The integrity of the RNA was verified by the determination of an optical density (OD) absorption ratio of OD_260nm_:OD_280nm_ between 1.8 and 2.0 and by electrophoresis with ethidium bromide staining on a 1% denaturing agarose gel. The total RNA (400 ng) was reverse‐transcribed to cDNA by using 4 U of Omniscript Reverse Transcriptase (Qiagen), according to the manufacturer's instructions. In the preliminary tests, the condition and the amount of the total RNA were compared when it was stored at −20°C in RNA later or at −80°C. It was confirmed that there was no significant difference between the two preservation methods.

### 2.6 Real‐time reverse transcription polymerase chain reaction analysis

Polymerase chain reaction (PCR) primers were designed by using the EMBL database (for CINC, COX‐2, Bcl‐2, and Bax) or were purchased (for IL‐1 beta and TNF alpha) from Nihon Gene Research Laboratory, Inc. (Sendai, Japan). Their sequences and the expected length of the PCR product are shown in Table 1. All the primers were tested by using conventional PCR and the PCR products were verified by sequencing. TITANIUM Taq DNA polymerase (BD Biosciences Clontech, Palo Alto, CA, USA) was used for a conventional PCR process. In brief, a master mix of the following reaction components (QuantiTect SYBR Green PCR kit; Qiagen) was prepared at the following final concentrations: 7.8 μL water, 10 μL Master Mix (containing 2.5 mmol/L Mg^2+^), 0.1 μL forward primer (0.5 µmol/L), and 0.1 μL reverse primer (0.5 µmol/L). The glass capillaries were filled with mixing fluid and 2 μL of PCR template that contained 300 ng of reverse‐transcribed total RNA was added. In order to ensure an accurate quantification of the desired product, at high‐temperature fluorescence, measurements were taken in the fourth segment of the PCR run. The elevated temperature that was used for the fluorescence acquisition results in the melting of non‐specific products, such as primer–dimmers, and in the elimination of non‐specific fluorescence signals. The following general real‐time PCR protocol was used: denaturation for 15 minutes at 95°C, 45‐55 cycles of a four‐segmented amplification and quantification program (the factor‐specific conditions are summarized in Table 2), a melting step of slow heating from 60°C to 99°C at a rate of 0.1°C/s and continuous fluorescence measurement, and a final cooling down to 40°C. Crossing point (CP) values were acquired by using the second derivative maximum method of the LightCycler software v. 3.3 (Roche Diagnostics, Mannheim, Germany). All the CPs of the 49 samples (n=6‐10 per group) per investigated factor were detected in one run to eliminate interassay variance. The real‐time PCR efficiencies were acquired by amplification of a standardized dilution series and the slopes were determined by using LightCycler Software 3.3 (Roche). The corresponding efficiencies (E) then were calculated according to the equation *E*=10^(−1/slope)^. The specificity of the desired products in the rat ovary was documented by using high‐resolution gel electrophoresis and an analysis of the melting temperature.

**Table 1:** Forward (For) and reverse (Rev) primer sequences (5′–3′), reverse transcription polymerase chain reaction product length, and reference of the investigated factors or of the corresponding accession number (Acc. No.) in the EMBL database

Primer	Sequence (5′–3′)	Length (bp)	Reference
CINC/gro	For ATGGTCTCAGCCACCCGCTCG Rev ACTTGGGGACACCCTTTAGC	289	Acc. No. NM_030845
COX‐2	For CAGGTCATCGGTGGAGAGGTGTATCC Rev GTTTGGAACAGTCGCTCGTCATCCC	230	Acc. No. AF233596
IL‐1 beta	For ACCTGTCCTGTGTGATGAAA Rev TGAAGTCAACTATGTCCCGA	234	Acc. No. M98820^a^
TNF alpha	For CAGACCCTCACACTCAGATCA Rev GTCCCTTGAAGAGAACCTGG	211	Acc. No. NM_012675^a^
Bcl‐2	For TTCCAGCCTGAGAGCAACCGAAC Rev TAGCGACGAGAGAAGTCATCCCC	164	Acc. No. L14680
Bax	For CAAGAAGCTGAGCGAGTGTCT Rev GGTTCTGATCAGCTCGGGCAC	238	Reference^30^
GAPDH	For TGGAGAAGGTGGGGCTCACCTG Rev CCACAACGGATACATTGGGGGTAGGAAC	417	Acc. No. NM_017008

a Nihon Gene Research Laboratories, Inc., Sendai, Japan. Bax, Bcl2‐associated X; CINC/gro, cytokine‐induced neutrophil chemoattractant; COX, cyclooxygenase; GAPDH, glyceraldehyde‐3‐phosphate dehydrogenase; IL, interleukin; TNF, tumor necrosis factor.

**Table 2:** Factor‐specific conditions for LightCycler real‐time polymerase chain reaction amplification and quantification of the investigated factors

Factor	Denaturation 15 seconds (°C)	Annealing 20 seconds (°C)	Elongation 72°C (seconds)	Cycle number
CINC/gro	94	63	13	50
COX‐2	94	65	10	50
IL‐1 beta	94	60	10	50
TNF alpha	94	52	10	50
Bcl‐2	94	66	10	55
Bax	94	67	12	55
GAPDH	94	65	20	50

Bax, Bcl2‐associated X; CINC/gro, cytokine‐induced neutrophil chemoattractant; COX, cyclooxygenase; GAPDH, glyceraldehyde‐3‐phosphate dehydrogenase; IL, interleukin; TNF, tumor necrosis factor.

### 2.7 Immunohistochemistry

Immunohistochemical staining also was performed on the tissue samples that had been taken from the rats at 6, 12, and 24 hours after the hCG injection. The ovaries that had been embedded in paraffin were sectioned (5 μm thick). The tissue sections were deparaffinized in xylene and dehydrated in a graded series of ethanol. The slides then were heated in an autoclave at 120°C for 5 minutes in a Tris‐HCl buffer (pH=7.4). An immunohistochemical analysis of COX‐2, IL‐1 beta, and TNF alpha was performed by using the labeled streptavidin–biotin method and the antigen–antibody complex was visualized by using the 3,3′‐dimaminobenzidine (DAB) solution. This process was performed with a LSAB+ Kit (DakoCytomation, Kyoto, Japan) and hematoxylin was used as the counterstain. Subsequently, the slides were mounted following dehydration in ethanol and xylene. Normal rabbit serum and normal goat serum were used as the negative controls.

### 2.8 Apoptosis detection by in situ analysis

DNA fragmentation was analyzed by the TUNEL method by using an apoptosis in situ detection kit (ApopDETEK; Enzo Life Sciences, Farmingdale, NY, USA) at three time points, according to the kit supplier's instructions. Briefly, the ovaries that had been embedded in paraffin were sectioned (5 μm thick). The tissue sections were deparaffinized in xylene, dehydrated in a graded series of ethanol, and covered by 1:20 diluted Proteinase K (DakoCytomation) in phosphate‐buffered saline for 5 minutes. The endogenous peroxidase activity was inhibited by quenching the samples for 10 minutes in 3% hydrogen peroxide. Thereafter, the labeling reaction was carried out by incubating the tissue sections with a buffer containing biotin‐dUTP and TdT for 45 minutes at 3°C. The tissues then were incubated for 60 minutes with a peroxidase–avidin complex and the apoptotic cells were visualized after reaction with the DAB solution.

### 2.9 Statistical analysis

The non‐parametric Mann‐Whitney *U*‐test was used to compare each mRNA expression level. The data were presented as the mean±SD from the mean and a *P*‐value of <.05 was considered to be statistically significant.

## 3 Results

The effect of co‐injection of 10 μg of anti‐rat CINC/gro or control normal serum with hCG into rats on ovulation was determined by analysis of the following parameters.

### 3.1 Number of oocytes

**Figure 1:**
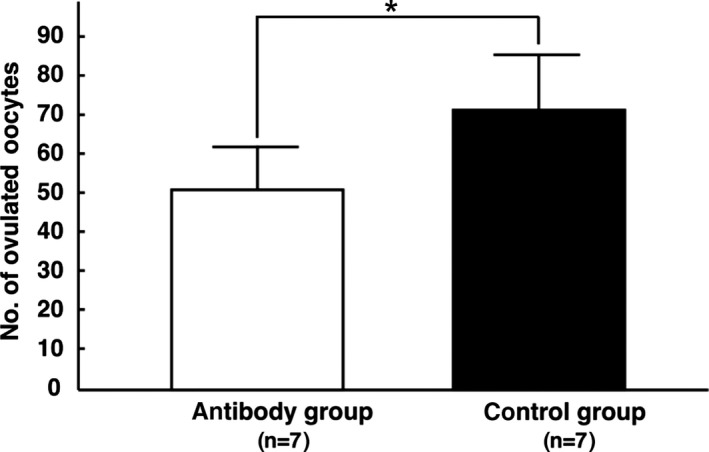
Cytokine‐induced neutrophil chemoattractant (CINC/gro) neutralization resulted in a reduced number of ovulated oocytes. The ovulated oocytes were obtained from the oviducts of rats 24 hours after the administration of hCG with or without the anti‐CIC/gro antibody. The collected oocytes were treated with hyaluronidase and counted under a microscope. *P<.05

Twenty‐four hours after the administration of the hCG, the number of ovulated oocytes in the antibody and control groups was 50±11.7 (n=7) and 72.9±12.5 (n=7), respectively (Figure 1). Thus, the total number of ovulated oocytes in the antibody group was significantly lower than that in the control group (*P*<.05).

### 3.2 Neutrophil infiltration

**Figure 2:**
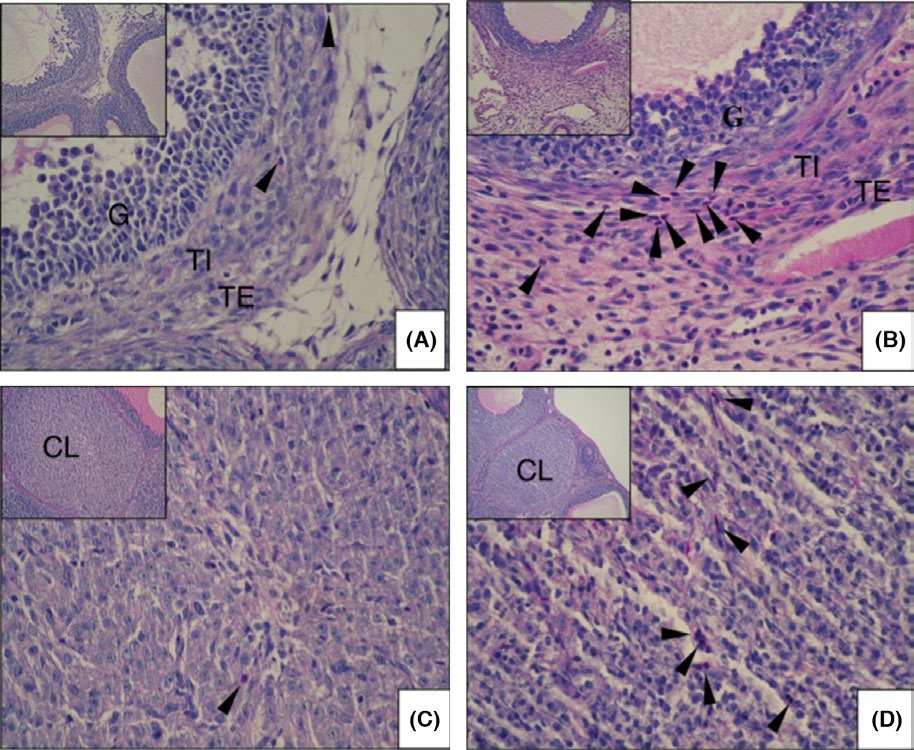
Effect of cytokine‐induced neutrophil chemoattractant (CINC/gro) neutralization on neutrophil infiltration. The ovarian tissues were immunohistochemically analyzed by periodic acid–Schiff staining following human chorionic gonadotropin administration. The arrow heads indicate neutrophils. After 12 hours of treatment, the number of neutrophils in the theca cell layer was strongly reduced in the (A) antibody group, as compared with the (B) control group. After 24 hours, the number of neutrophils in the corpus luteum was strongly reduced in the (C) antibody group, as compared with the (D) control group. The large slides are shown at ×400 magnification and the insets at the top left of the large slides are shown at ×200 magnification. CL, corpus luteum; G, granulosa cells; TE, theca externa; TI, theca interna

Neutrophil infiltration into the ovaries was histologically analyzed by PAS staining of the ovarian sections that had been taken at 6, 12, and 24 hours after the administration of the hCG with or without the anti‐CINC/gro antibody. At 6 hours after the hCG treatment, there was no significant difference between the antibody group and the control group (data not shown). However, after 12 hours, a clear difference between the two groups was seen: there were many granulocytes in the theca layer in the control group (Figure 2B), but only a few granulocytes in the theca layer of the antibody group (Figure 2A). This difference persisted up to the 24 hours time point. At 24 hours, many granulocytes had infiltrated into the corpus luteum in the control group (Figure 2D), whereas granulocyte migration was clearly reduced in the antibody group (Figure 2C).

### 3.3 Messenger RNA expression of cytokine‐induced neutrophil chemoattractant, cyclooxygenase‐2, interleukin‐1 beta, TNF alpha, bcl‐2, and bcl2‐associated X

**Figure 3:**
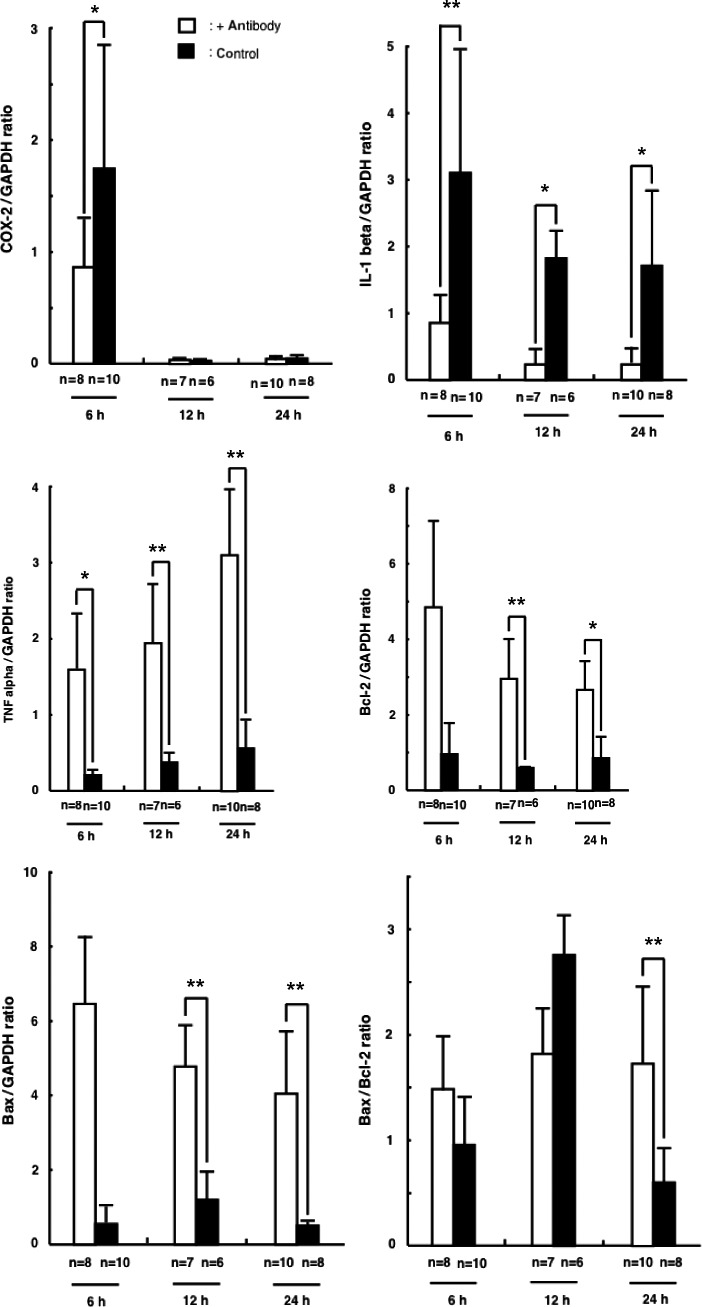
Changes in the messenger RNA (mRNA) expression of a range of proteins following human chorionic gonadotropin treatment with or without anti‐cytokine‐induced neutrophil chemoattractant antibody. The mRNA expression of the indicated proteins was examined by real‐time polymerase chain reaction by using the LightCycler. Each mRNA expression was normalized by using glyceraldehyde‐3‐phosphate dehydrogenase (GAPDH) mRNA expression and the values that are shown are mRNA: GAPDH mRNA ratios. The 6 hours measurement of cyclooxygenase‐2 and all of the time point measurements of interleukin‐1 beta were significantly decreased in the antibody group, compared with the control group. The levels of tumor necrosis factor (TNF) alpha, Bcl‐2, and Bcl2‐associated X (Bax) were higher in the antibody group than in the control group at all time points and these values were significantly different for TNF alpha at all time points and for Bcl‐2 and Bax at the 12 and 24 hours time points. The increase in the ratio of Bax/Bcl‐2 was only significantly different from the control group at the 24 hours time point (*P*<.01). *P<.05 and **P<.01

The effect of the anti‐CINC/gro antibody on the mRNA expression of a range of proteins that are known to be involved in ovulation was assayed by using a real‐time PCR method. There was no significant difference between the CINC mRNA expression in the antibody group and the control group at any time point after hCG administration (data not shown). The level of COX‐2 mRNA expression was significantly (*P*<.05) reduced in the antibody group at 6 hours after hCG administration, when compared with the control group. At this time point, the level of COX‐2 mRNA expression in the presence of the anti‐CINC/gro antibody was 45% that of the control group. However, at the other time points that were tested, there was no significant difference in the level of COX‐2 expression between the two groups. The expression of IL‐1 beta was down‐regulated in the antibody group at all time points, compared to the control group (*P*<.05 at 6 hours and *P*<.01 at 12 and 24 hours). The level of IL‐1 beta mRNA expression of the antibody group was .28‐fold that of the control group at 6 hours after hCG administration, but was lower at later time points (0.16 and 0.19 that of the control at 12 and 24 hours, respectively). The mRNA expression of TNF alpha was significantly increased at all time points in the antibody group, compared to the control group (*P*<.05 at 6 hours and *P*<.01 at 12 or 24 hours). Although the TNF alpha mRNA expression did increase over time in the control group, the level in the antibody group exceeded that in the control group at all time points (6 hours, 7.5‐fold increase vs the control; 12 hours, 6.4‐fold increase vs the control; 24 hours, 5.2‐fold increase *vs* the control). The mRNA expression of Bcl‐2 and Bax in the antibody group also exceeded that of the control group at all time points; however, the differences in expression were only significant at later time points (12 and 24 hours, *P*<.01) and not at 6 hours after hCG administration. The Bax/Bcl‐2 mRNA ratio was calculated at all time points. This ratio was significantly higher (3.3‐fold higher compared to the control, *P*<.01) in the antibody group than in the control group at 24 hours after hCG administration (Figure 3).

### 3.4 Expression of the cyclooxygenase‐2, interleukin‐1 beta, and TNF alpha proteins

**Figure 4:**
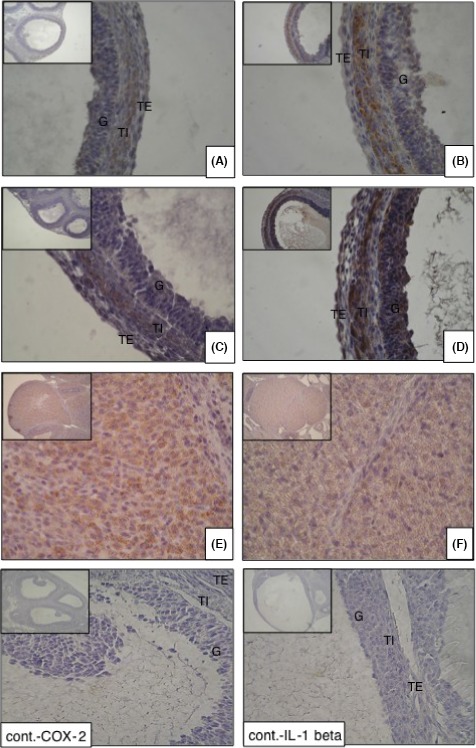
Effect of cytokine‐induced neutrophil chemoattractant (CINC/gro) antibody on the expression of cyclooxygenase (COX)‐2, interleukin (IL)‐1 beta, and tumor necrosis factor (TNF) alpha protein. The expression of COX‐2, IL‐1 beta, and TNF alpha protein was examined by immunohistochemical staining. After 6 hours of human chorionic gonadotropin treatment, the COX‐2 protein was expressed at the theca layer in both the (A) antibody group and the (B) control group. Similarly, the IL‐1 beta protein was observed at the theca layer in both the (C) antibody group and the (D) control group. The expression of these proteins was more clearly observed in the antibody group than in the control group. The most intense staining of TNF alpha was observed in the corpus luteum after the 24 hours treatment and its expression was stronger in the (E) antibody group than in the (F) control group. G, granulosa cells; TE, theca externa; TI, theca interna

The expression of the COX‐2, IL‐1 beta, and TNF alpha proteins was assessed by immunohistochemical staining of the ovaries. The immunohistochemical staining of COX‐2 and IL‐1 beta was observed in the theca cell layer at 6 hours after hCG administration in both the antibody group (Figure 4A,C, respectively) and the control group (Figure 4B,D, respectively). However, the staining of COX‐2 and IL‐1 beta was more intensive in the control group than in the antibody group. The COX‐2 and IL‐1 beta staining was observed at the later time points (12 and 24 hours) in the granulose lutein cells in both groups and staining was similar in the antibody and control groups. In contrast, although as expected, the TNF alpha expression was observed in the theca cell layer at 6 hours and in the granulose lutein cells at 12 and 24 hours after hCG administration, the intensity of the TNF alpha staining was stronger in the antibody group (Figure 4E) than in the control group (Figure 4F) only at the 24 hours time point.

### 3.5 Atresia and apoptosis of the follicles

**Figure 5:**
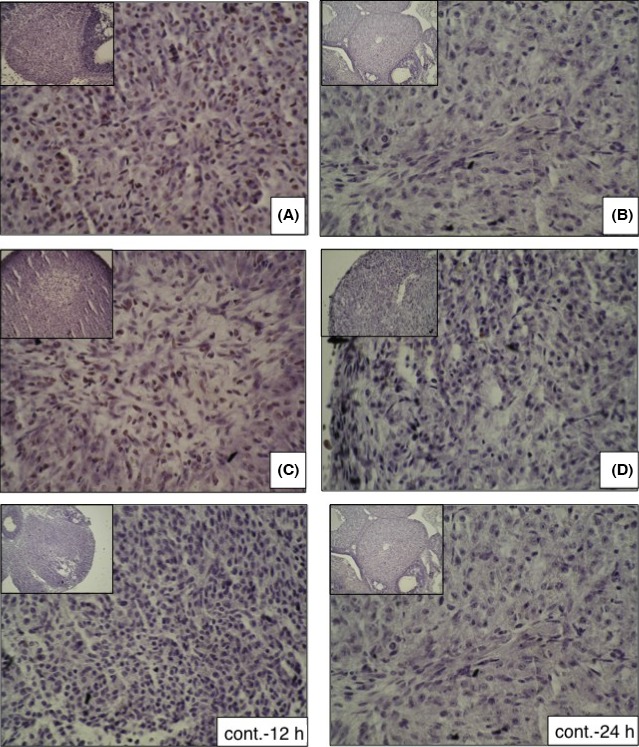
Effect of cytokine‐induced neutrophil chemoattractant (CINC/gro) antibody on atresia and apoptosis. Atresia and apoptosis were histologically investigated following treatment. After 12 hours, some premature luteolysis was observed in both the (A) antibody group and the (B) control group, but there appeared to be higher atresia in the antibody group than in the control group. After 24 hours, a high number of corpus lutea was observed in both the (C) antibody group and the (D) control group. There was no terminal deoxynucleotidyl transferase‐mediated dUTP nick end labeling‐positive cell in the control group after either 12 or 24 hours

In this model, ovulation occured between 12 and 15 hours after hCG injection. Thus, at 12 hours after hCG administration, the ovarian follicle that was filled with the real ingredient that did not fill the fluid resulted in follicular atresia through the formation of a premature luteal body for incomplete development. At 12 hours after hCG administration, it was observed that the majority of the follicles in the ovarian sections had not yet ruptured, though a small number of follicles had progressed to atresia. Therefore, the follicles were examined at time points that started 12 hours after hCG administration. At the 12 hours time point, the antibody group (Figure 5A) showed greater progression to atresia than the control group (Figure 5B). Furthermore, apoptotic cells were detected in the antibody group by using the TUNEL method. At 24 hours after hCG administration, the degree of follicular atresia was similar in both groups; however, apoptotic cells were only detected in the antibody group (Figure 5C) and not in the control group (Figure 5D).

## 4 Discussion

In the previous study, a reduction of the ovulation rate in the rat by neutrophil‐depleting antibody was demonstrated^26^ and it also was demonstrated that the CINC/gro‐1 antibody decreased the number of ovulated oocytes. The CINC/gro‐1 antibody also influenced the mRNA expression of COX‐2, IL‐1 beta, and TNF alpha, resulting in a decrease in COX‐2 and IL‐1 beta mRNA expression and an increase in TNF alpha mRNA expression, and it was known that PMSG–hCG stimulation only increased COX‐2 mRNA expression at 4 hours in mice.^12^ Immunohistochemical staining confirmed that the expression of these proteins was changed accordingly. This study's data show that apoptosis of the premature follicle is controlled by CINC/gro via its inhibition of TNF alpha expression. Over‐expression of TNF alpha is considered to trigger an increase in the Bax/Bcl‐2 ratio, which favors apoptosis. Therefore, the increase in TNF alpha expression that is induced by the anti‐rat CINC/gro‐1 antibody would be expected to result in the continuous induction of apoptosis.

The ovulation rate is reduced by COX‐2 suppression and this phenomenon can be inhibited by supplying prostaglandin E_2_ or IL‐1 beta.^13^ However, it was unknown whether CINC/gro or COX‐2 took precedence in this process. It is confirmed that COX‐2 expression is suppressed by the administration of the anti‐rat CINC/gro‐1 antibody. It was demonstrated elsewhere that CINC/gro is present in the theca layer in the rat ovary just prior to ovulation.^5^ It is demonstrated that CINC/gro plays an important role in the preovulatory phase when COX‐2 expression is suppressed by the administration of anti‐CINC/gro antibody, although the decrease is not down to zero in ovulation number.

The CINC/gro is a chemokine and it has been reported that chemokines are induced by cytokines, such as IL‐1 beta and TNF alpha.^2^ However, there have been no previous reports indicating that the suppression of CINC/gro results in decreased IL‐1 beta expression or in increased TNF alpha expression. An earlier study showed that the administration of IL‐1 beta and TNF alpha induced an increase in CINC/gro expression.^5^ Furthermore, although the levels of IL‐1 beta, TNF alpha, and CINC/gro all were increased after hCG administration, the IL‐1 beta and TNF alpha levels peaked at 4 hours and the CINC/gro levels peaked at 6 hours after injection. In the present study, it is demonstrated that antibody suppression of CINC/gro decreased IL‐1 beta expression at 6, 12, and 24 hours after hCG treatment, as compared to the controls. These results show that IL‐1 beta expression is dependent on CINC/gro and vice versa. In contrast to its effect on IL‐1 beta, the anti‐CINC/gro antibody induced an increase in TNF alpha expression, as compared to the control. These phenomena are related to a decrease in the ovulation rate and lead to follicular atresia and apoptosis.^27^, ^28^

The TNF alpha is an important element for follicular atresia and apoptosis.^20^, ^28^, ^29^ In this study, apoptosis by TUNEL staining and by the expression ratio of Bax and Bcl‐2 mRNAs was assessed. The results suggest that the suppression of neutrophil infiltration promotes apoptosis. Thus, at all the time points that were tested after hCG administration (6, 12, and 24 hours), the mRNAs of TNF alpha, Bax, and Bcl‐2 were significantly higher in the presence, than in the absence, of the CINC/gro antibody. However, the increase in the Bax/Bcl‐2 ratio in the presence of the antibody was only significantly higher than the control at 24 hours after hCG treatment. These data suggest that neutrophil infiltration plays a role in follicular atresia and apoptosis by controlling the expression of TNF alpha, Bax, and Bcl‐2.^18^, ^21^, ^27^ The over‐expression of TNF alpha, an increase in the Bax/Bcl‐2 ratio, and an increase in TUNEL‐positive cells that are induced by the CINC/gro antibody all suggest that CINC/gro controls the apoptosis of the follicles via TNF alpha expression.

The above data show that ovulation in rats is accelerated by neutrophil infiltration into the theca layer that is induced by the expression of CINC/gro and that CINC/gro and IL‐1 beta synergize to bring about ovulation. This study's data further suggest that the expression of CINC/gro suppresses TNF alpha expression and thereby prevents the follicles from undergoing atresia and apoptosis.

## Disclosures

*Conflict of interest*: The authors declare no conflict of interest. *Human studies*: This article does not contain any study with human participants that was performed by any of the authors. *Animal studies*: All the institutional and national guidelines for the care and use of laboratory animals were followed.
